# What are our health expectations in a pandemic?

**DOI:** 10.1111/hex.13052

**Published:** 2020-03-30

**Authors:** Parisa Aslani

**Affiliations:** ^1^ The University of Sydney School of Pharmacy Faculty of Medicine and Health Camperdown NSW Australia

For weeks, we were warned of a potential coronavirus disease 2019 (COVID‐19) pandemic,^1^ and on 11th March 2020, the World Health Organization announced it as a pandemic.^2^ Questions continue to be asked about how well the health‐care systems around the world are equipped to cope. From an individual and community perspective, what are our health expectations in a pandemic?

At times like these, people seek information through all possible channels, from their neighbours, family members and friends, to the media, the Internet, health‐care professionals and government organizations. This thirst for information is to address their knowledge gaps, inform their understanding of current events and future developments, and, importantly, help determine how vulnerable they are to contracting COVID‐19 and what actions need to be taken for prevention and treatment. We expect accurate, timely and reliable information provided in a way that can be quickly accessed and easily understood. In this digital age, information has become a commodity that we expect to have ready access to, and receive much more frequently than in the past, via reputable news media and their online and social networking sites. We expect to receive accurate and reliable information from national and international organizations we trust, and, in turn, we expect that these organizations have received accurate and trustworthy data to report. We certainly expect the truth, but understand that up‐to‐date information may not always be possible, considering the time lag in data collection and data reporting, and the variable resources available globally to collate data. Lack of information, misinformation and inaccurate information can lead to hysteria and fuel behavioural outcomes such as panic buying.^3^ A well‐informed public is critical at a time when the community is impacted by a viral outbreak.

We expect to be informed about prevention, and what we can do as individuals to help slow down the spread of the virus and subsequent infection within our communities. This goes beyond simply being told to practise good hygiene—people need to know exactly what good hygiene entails. For example, we know we must wash our hands, but do we know when, how frequently, with what and for how long? If we are expecting to educate people, then the information must be understandable and actionable. If 20 seconds of hand washing with soap is part of practising good hygiene,^4^ then this is the information that should be effectively communicated. Better still, it should be linked with an easily remembered action, such as singing a song that lasts 20 seconds.^4^ People also need to know how effective preventative measures are. An understanding of the rationale for behaviour and its associated benefits and impact is more likely to motivate people to adopt the behaviour.

When faced with the unknown and unprecedented, fear and concern would be expected within the community. We expect to know what happens to a person who becomes infected with COVID‐19. Clear and consistent information about the impact of an infection in otherwise healthy individuals, as well as in children, the elderly and those with underlying health problems is vital when people are facing a pandemic. With nearly 180 000 people (as of 17 March 2020) infected globally,^5^ there should be enough data to accurately provide information about symptoms, severity and duration of the infection; as well as full recovery and mortality rates; characteristics of those recovering and those who have succumbed to the virus; cause(s) of death; and potential for reinfection.

Systems at a local, national and international level must work together to combat such novel threats and support the health and wellbeing of populations worldwide. We look to our governments to have COVID‐19 action plans that would protect us from a pandemic. And undoubtedly, we want well‐resourced health‐care systems that enable timely and appropriate health‐care delivery, capable of effectively treating us should we end up in the infectious diseases ward of a hospital. We expect our health‐care workers to be supported and looked after so that they remain healthy to look after those infected. In short, we expect and hope to be filled with a sense of security when we are on the brink of a pandemic. This sense of security can come from knowledge that we are receiving timely, accurate and reliable information from trustworthy sources that are ‘louder’ than those sources providing biased, inaccurate and incorrect information.

Our efforts to overcome the COVID‐19 outbreak have included research in identifying and better understanding the virus, developing a vaccine and identifying drug targets. It has also led to the development of professional guidelines, public health information and initiatives. Promisingly, we have seen governments and health‐care systems mobilized into action to prevent the spread of the virus—this has been the primary goal in the past three months. However, has there been a person‐centred approach to the actions taken to date? Has there been equity, between the public and those within the government and health‐care systems, in the processes implemented to ensure that the public are involved in the decisions made and plans prepared? How can we better engage the public in preparing for, and to act, in the face of a pandemic?

The motivation to adopt appropriate public health behaviour is high—people do not want to contract the disease, which could be fatal in some cases, nor do they want to be restricted in their movements and contacts, or be in quarantine, whether forced or self‐imposed. In today's world, where our economies are interlinked and travelling for leisure and business is an important part of our lives, global citizenship will underpin our responses and expectations. We continue to expect accurate, trustworthy and consistent information we can understand and act on.
